# Complete Genome Sequence of *Psychrobacillus* sp. Strain INOP01, a Phosphate-Solubilizing Bacterium Isolated from an Agricultural Soil in Germany

**DOI:** 10.1128/mra.00207-22

**Published:** 2022-04-04

**Authors:** Akane Chiba, Manuela Peine, Susanne Kublik, Christel Baum, Michael Schloter, Stefanie Schulz

**Affiliations:** a Research Unit Comparative Microbiome Analysis, Helmholtz Zentrum München, Neuherberg, Germany; b Crop Physiology, TUM School of Life Sciences, Technical University of Munich, Freising, Germany; c Soil Science, Faculty of Agricultural and Environmental Sciences, University of Rostock, Rostock, Germany; d Soil Science, TUM School of Life Sciences, Technical University of Munich, Freising, Germany; University of Delaware

## Abstract

We report the complete genome sequence of the phosphate-solubilizing bacterium *Psychrobacillus* sp. strain INOP01, isolated from an agricultural field in Rostock, Germany. In addition to its phosphate-solubilizing ability, the genome contains genes coding for proteins involved in phosphate (P) acquisition from various sources.

## ANNOUNCEMENT

We isolated *Psychrobacillus* sp. strain INOP01 from an agricultural field in Rostock, Germany (54°3′41.47″N, 12°5′5.59″E), where no P fertilizer has been applied since 1998 (1). The isolation was performed in May 2016 from one gram of fresh soil, by serial dilution plating (2) onto Pikovskaya agar plates containing tricalcium phosphate (10 g L^−1^) as the sole P source (3). The plates were incubated under aerobic conditions at 28°C until halo zones appeared, indicating phosphate solubilization. The strain was identified by comparing the whole genome to other type strains using the Type Strain Genome Server (TYGS) (https://tygs.dsmz.de/) (4), which did not reveal close relationships between our strain and those deposited in the server. Therefore, a phylogenetic analysis using the extended TYGS 16S rRNA gene pipeline was conducted.

For genome sequencing, high-molecular-weight genomic DNA was extracted using the Genomic-tip 20/G kit (Qiagen, Hilden, Germany) from overnight cell cultures grown in standard nutrient broth (15 g/L peptone, 3 g/L yeast extract, 6 g/L sodium chloride, 1 g/L glucose; pH 7.5 ± 0.2) at 30°C. The obtained DNA was sheared to approximately 10 kb using g-TUBEs (Covaris, Inc., Woburn, MA) without additional size selection. Genome sequencing was conducted on the PacBio Sequel platform (Menlo Park, CA). Library preparation was performed following the PacBio protocol “Procedure & Checklist - Preparing Multiplexed Microbial Libraries Using SMRTbell Express Template Prep Kit 2.0” (product number 101-696-100, v6 [March 2020]). The library was loaded onto two single-molecule real-time (SMRT) cells 1M v3 at concentrations of 3 pM and 6 pM, respectively, using diffusion loading according to the manufacture’s recommendations. SMRT sequencing was performed on the Sequel System SMRT Link v8.0.0.80529 with 120 min immobilization and 120 min preextension, followed by a 600-min movie time per SMRT cell.

After demultiplexing, a total of 362,412 subreads (mean subread length, 2,792 bp) were assembled using the Hierarchical Genome-Assembly Process 4 pipeline embedded in SMRT Link v9.0.0.92188 with default parameters. The mean coverage was 375-fold. The genome of *Psychrobacillus* sp. INOP01 contained one chromosome scaffold, which was circularized using the parameter --merge_min_length_merge 1000 in Circlator v1.5.5 (5). Coding sequences (CDSs) and rRNA and tRNA genes were scanned and annotated using the NCBI Prokaryotic Genome Annotation Pipeline (PGAP) (6). The genome was functionally annotated using the RASTtk (Rapid Annotations using Subsystems Technology toolkit) v1.073 (7) in KBase (https://www.kbase.us/).

The circularized genome had a size of 4,301,978 bp, contained 4,077 predicted CDSs, and had a GC content of 36.4%. No additional plasmids were found. A total of 30 rRNA genes, 74 tRNAs, and 1 transfer-messenger RNA were annotated using PGAP. Based on the RASTtk annotation, genes coding for proteins involved in phosphonate solubilization (8), the ATP-dependent phosphate uptake system (9), alkaline phosphatase (EC 3.1.3.1), and the two-component regulatory system of the Pho regulon (10) were detected. This indicates the potential of *Psychrobacillus* sp. INOP01 to use versatile phosphorus sources.

### Data availability.

The complete annotated genome sequence of *Psychrobacillus* sp. INOP01 has been deposited at GenBank under the accession number GCA_018140925.1. The raw reads have been deposited under the SRA accession number SRR14181225 and the BioProject accession number PRJNA720408.
